# Evidence Supporting the Hypothesis That Inflammation-Induced Vasospasm Is Involved in the Pathogenesis of Acquired Sensorineural Hearing Loss

**DOI:** 10.1155/2019/4367240

**Published:** 2019-11-06

**Authors:** Michael Eisenhut

**Affiliations:** Luton and Dunstable University Hospital NHS Foundation Trust, Lewsey Road, Luton LU40DZ, UK

## Abstract

Sensorineural hearing loss is mainly acquired and affects an estimated 1.3 billion humans worldwide. It is related to aging, noise, infection, ototoxic drugs, and genetic defects. It is essential to identify reversible and preventable causes to be able to reduce the burden of this disease. Inflammation is involved in most causes and leads to tissue injury through vasospasm-associated ischemia. Vasospasm is reversible. This review summarized evidence linking inflammation-induced vasospasm to several forms of acquired sensorineural hearing loss. The link between vasospasm and sensorineural hearing loss is directly evident in subarachnoid haemorrhage, which involves the release of vasoconstriction-inducing cytokines like interleukin-1, endothelin-1, and tumour necrosis factor. These proinflammatory cytokines can also be released in response to infection, autoimmune disease, and acute or chronically increased inflammation in the ageing organism as in presbyacusis or in noise-induced cochlear injury. Evidence of vasospasm and hearing loss has also been discovered in bacterial meningitis and brain injury. Resolution of inflammation-induced vasospasm has been associated with improvement of hearing in autoimmune diseases involving overproduction of interleukin-1 from inflammasomes. There is mainly indirect evidence for vasospasm-associated sensorineural hearing loss in most forms of systemic or injury- or infection-induced local vascular inflammation. This opens up avenues in prevention and treatment of vascular and systemic inflammation as well as vasospasm itself as a way to prevent and treat most forms of acquired sensorineural hearing loss. Future research needs to investigate interventions antagonising vasospasm and vasospasm-inducing proinflammatory cytokines and their production in randomised controlled trials of prevention and treatment of acquired sensorineural hearing loss. Prime candidates for interventions are hereby inflammasome inhibitors and vasospasm-reducing drugs like nitric oxide donors, rho-kinase inhibitors, and magnesium which have the potential to reduce sensorineural hearing loss in meningitis, exposure to noise, brain injury, arteriosclerosis, and advanced age-related and autoimmune disease-related inflammation.

## 1. Introduction

Vasospasm is a consistent feature of all forms of cerebral inflammation including forms caused by infections like bacterial meningitis, cerebral malaria, and sterile vascular inflammation as detectable in diabetic ketoacidosis and brain injury [1, 2]. Vasospasm is hereby mediated by depletion of nitric oxide and direct effects of interleukin-1 (IL-1), tumor necrosis factor (TNF), and endothelin-1. Cerebral vasospasm has been associated with cerebral ischemia and subsequent neurological deficits. The cochlear hair cells are supplied almost exclusively by a single terminal artery, which is the labyrinthine artery also called the spiral modiolar artery, a branch of the anterior inferior cerebellar artery (AICA), which terminates radially in the lateral cochlear wall, thereby forming the stria vascularis. Cochlear hair cells have high oxygen consumption and poor tolerance to hypoxia. In “vasospasm-related” sensorineural hearing loss (SNHL), which is caused by ischemia, the pattern should be a sudden loss of hearing across several frequencies, which in most cases would start or be exclusively unilateral because a synchronicity of vasospasm occurring at exactly the same time in both ears is unlikely unless caused by a factor which acts equally on both labyrinthine arteries like an ototoxic drug or equal exposure to a source of noise. Trauma, infection, autoimmune-, or arteriosclerosis-related transient vascular stenosis is likely due to an asymmetrical exposure of the supply artery to cytokines causing vasospasm. The hearing loss should be sensorineural but reversible in a percentage of patients indicating that not hair cell loss but temporary hair cell dysfunction secondary to temporary ischemia as found in a transient vasospasm is responsible in some cases. In this review, I have attempted to summarize the evidence for a link between inflammation-induced vasospasm and acquired SNHL as well as approaches to prevention and treatment.

## 2. Methods

### 2.1. Inclusion Criteria

Included were the following studies:

- Reports providing evidence relating to conditions associated with inflammation and SNHL and/or a focus on a potential association between inflammation-induced vasospasm and SNHL. As indicator of vasospasm was in the interpretation hereby taken that a proportion of people affected by a condition associated with SNHL showed laterality/ and or reversibility of the SNHL.
- Both animal and human studies.
- All studies reporting randomised controlled trials of anti-inflammatory or vasospasm-relieving drugs in prevention and/or treatment of SNHL.
- Studies which reported on the pathophysiology of cytokine-induced hearing loss or vasospasm.

### 2.2. Exclusion Criteria

Excluded were the following studies:

- Studies in which patients with conductive hearing loss were investigated.
- Studies not reported in English language.
- Case reports on effect of treatment modalities on SNHL.
- Nonrandomised studies with regard to treatment trials in humans.
- Studies on compromise of vascular supply by arterial wall thickening, e.g., in vasculitis and endoluminal stenosis like in subendothelial atherosclerotic proliferation or in obstruction of the vascular lumen by embolism.

### 2.3. Database Search

Searches using the following keywords were carried out in September 2019: (i) “hearing loss” and “vasospasm” in the PubMed database and (ii) “sensorineural hearing loss” in the Cochrane Library database.

**Figure 1:**
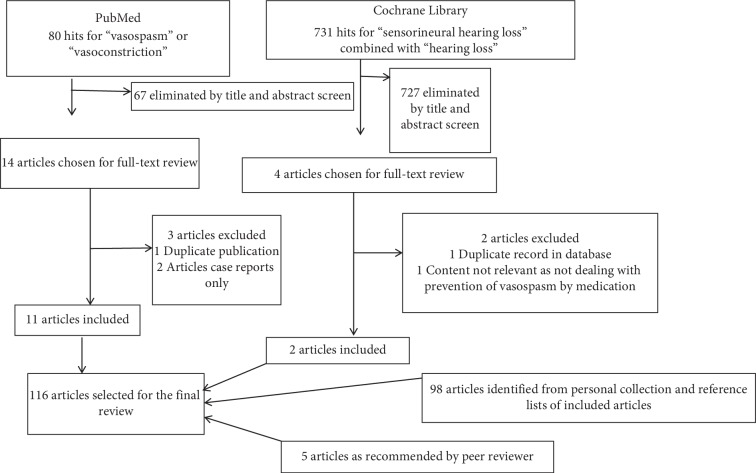
Flow chart of literature search.

References of relevant articles were searched and incorporated where indicated. For flow chart of literature search, see Figure 1.

### 2.4. Categorisation of Evidence of a Link between SNHL and Inflammation-Induced Vasospasm

In the descending order of strength of a potential causal association of inflammation-induced vasospasm and SNHL, conditions were categorized into 3 categories:

1. “Direct evidence” for inflammation-associated vasospasm causing SNHL studies where there was evidence of vasospasm on in vivo dynamic imaging like Doppler flow studies in patients with conditions with SNHL more than in patients without the same conditions. Evidence regarding vasoconstrictors and treatment with ant-inflammatory agents and vasodilators was similarly labelled if based on a model with direct evidence.
2. “Features consistent with inflammation-associated vasospasm-induced SNHL” and the associated treatment addressing and/or leading to a change in those features.
3. “Conditions associated with increased risk of inflammation associated with vasospasm-induced SNHL.” All forms of inflammation involving vasoconstriction causing cytokines were regarded as putting the cochlear perfusion at risk of vasospasm-induced compromise.

## 3. Results

### 3.1. Inflammatory and/or Vasospastic Aspects of Clinical Entities Associated with Hearing Loss

#### 3.1.1. Sudden Hearing Loss

Direct evidence for a vasospasm as etiological factor in acquired SNHL is the analysis of data from children with sudden SNHL where influence of noise exposure, medication and drugs including illicit substances, and exposure to chemicals at the work place which could cause temporary hair cell dysfunction is much less likely. A pooled analysis of 226 cases (ears) of sudden SNHL in children from two centres in China demonstrated 85% of children had unilateral hearing loss, of which the largest proportion was profound (51%). The hearing loss was reversible to a variable extent (including any form of recovery) in 48.6% [3, 4], and there was no dominance of a side. Evidence has emerged that microvascular dysfunction is associated with sudden onset SNHL. Microvascular dysfunction was hereby measured using videocapillaroscopic examination of the nailfold, measuring the capillary density (CD) and postocclusive reactive hyperaemia (PORH) values. Patients with sudden onset SNHL had significantly lower CD and PORH values [5]. Surgical induction of vasospasm in the labyrinthine artery has been shown to cause reversible SNHL [6].

#### 3.1.2. Autoimmune Diseases

The hearing loss found associated with autoimmune-induced inflammation has features consistent with inflammation-associated vasospasm-induced sensorineural hearing loss. The hearing loss in autoimmune-induced inflammation is potentially reversible, and anti-inflammatory treatment can prevent and treat SNHL [7].

Autoimmune diseases like systemic lupus erythematosus and Crohn's disease which induce overproduction of tumor necrosis factor (TNF) and rarer diseases like the cryopyrin-associated periodic syndromes with inflammasome activation and subsequent overproduction of interleukin-1(IL-1) like CINCA syndrome and Muckle–Wells disease have been associated with SNHL [8, 9]. The blockage of TNF with anti-TNF antibody constructs (for Crohn's disease) and anti-IL-1 (diseases with inflammasome activation) has been shown to reverse the hearing loss [10, 11]. This is against what would be permanent damage to hair cells or neuronal damage involved in hearing loss or apoptosis and favours a reversible cause of immune-mediated hearing loss like vasospasm.

#### 3.1.3. Bacterial Meningitis

In bacterial meningitis, severe inflammation of the meninges is observed which should, if there was a link between inflammation and SNHL lead to SNHL. Hearing loss is indeed observed in all forms of bacterial meningitis [10, 11].

In a prospective study of 236 children with meningitis using brainstem auditory evoked responses, 38 were detected with hearing loss in the acute (early) phase of meningitis; among these, 32 were available for follow-up, and within the latter group, 22 recovered normal hearing, i.e., reversibility was seen in 68.7% of the cases available for documentation. The hearing loss was unilateral in 21 of the 38 children with hearing loss detected in the acute meningitis phase (= 55.3%). The 21 unilateral HL cases among 236 children with bacterial meningitis amounted therefore to 9% [12].

An analysis of risk factors for SNHL in patients with bacterial meningitis identified focal neurological signs, as found in localized vasospasm, as an associated feature but not general severity of sepsis (see Table 1) [10]. Subsequent studies conducted in Malawi, Kenya, and Pakistan confirmed the association of SNHL with focal neurological deficits including cranial nerve palsies [13, 14]. A recent review article [1] highlighted that features of vasospasm were detected in the form of arterial stenosis in bacterial meningitis in a case of *Haemophilus influenzae* meningitis [15], whereas it was Greitz who was the first who detected evidence for vascular stenosis in the form of local vasoconstriction at the base of the brain in cases of *Mycobacterium tuberculosis* meningitis [16].

A prospective investigation of adults with bacterial meningitis by transcranial Doppler sonography related the degree of arterial narrowing to outcome including focal cerebral ischemic deficits, which were more frequent in patients with cerebral blood flow velocity >210 cm/s compatible with vasospasm [17].

Other investigations confirmed features of cerebral vasospasm in bacterial meningitis. Patients with features of vasospasm had significantly increased concentrations of IL-1 and IL-6 in the CSF. Underlying mechanisms have been explored in a rat model, where cerebral blood flow as measured by injection and recovery of microspheres from the brain tissue was found to be reduced in pneumococcal meningitis. The cerebral blood flow could be restored by the endothelin-1 antagonist bosentan with a reduction in cortical cerebral injury. Strong evidence of a protective role of the vasodilator nitric oxide against vasospasm in bacterial meningitis was derived from an animal model of neonatal meningitis where inhibition of NO synthase worsened outcome in some models of bacterial meningitis, leading to local cerebral ischemia [18, 19]. The findings from these studies highlight that it is not simply the level of cytokines causing sepsis but a specific cytokine profile and the response of the body to it. Steroids have been shown to reduce the risk of development of SNHL in bacterial meningitis. They reduced the risk of death (RR: 0.46, 95% CI: 0.24 to 0.88; 2 studies, *N* = 132; very low-quality evidence) but did not have a significant effect on the number of infants with SNHL at two years (RR: 1.80, 95% CI: 0.18 to 18.21; one study, *N* = 38 participants, low-quality evidence). In one trial, dexamethasone reduced the likelihood of hearing loss at 4 to 10 weeks after discharge (RR: 0.41, 95% CI: 0.17 to 0.98; one study, *N* = 59 participants, low-quality evidence). There was no beneficial effect of corticosteroid therapy in low‐income countries [20–22].

All these lines of evidence revealed features consistent with inflammation-associated vasospasm-induced SNHL but no direct evidence for a link between the hearing loss observed and vasospasm of blood supply of the cochlea.

#### 3.1.4. Head or Brain Injury

A recent systematic review of 13 studies encompassing 773 patients found that the studies with the highest level of evidence reported a transient or chronic hearing loss of at least 10–15 dB across a range of frequencies in as many as 58% of patients with traumatic brain injury without bone fracture [22].

Vasospasm has hereby previously been associated with brain injury and was more severe in patients with associated features of a systemic inflammatory response [1].

In subarachnoid haemorrhage, vasospasm has been directly linked to hearing loss [23, 24]. The underlying mechanism appears to be free haemoglobin from the extravasated blood scavenging the vasodilator nitric oxide and inducing the vasoconstricting cytokine IL-1 via inflammasome activation. However, traumatic head injury is a condition associated with increased risk of inflammation-associated vasospasm-induced sensorineural hearing loss. Subarachnoid haemorrhage has features consistent with inflammation-associated vasospasm-induced sensorineural hearing loss.

A retrospective case control study of neonatal brain injury including 58 infants with SNHL and comparing them with 116 controls without hearing loss established that cerebral infarction was significantly increased in infants with SNHL compared to normal hearing controls [25]. Cerebral infarction is a feature of vaso-occlusion and may be related to vasospasm in the neonatal period. Cerebral vasospasm may occur in association with intraventricular haemorrhages which were previously associated with vasospasm-induced cerebral injury via haem released from haemoglobin, scavenging the vasodilator nitric oxide and stimulating production of the vasospasm-inducing interleukin-1 by inflammasome activation [26].

#### 3.1.5. Noise-Induced Trauma

The labyrinthine artery is known to constrict during and after noise overstimulation [27]. TNF-*α* inhibition using etanercept prevented noise-induced hearing loss by improvement of cochlear blood flow in vivo [28, 29]. Prophylactic treatment with the calcium antagonist nimodipine was unsuccessful in prevention of noise-associated SNHL pointing to a calcium-independent effect of TNF on vasoconstriction [30]. For noise-induced hearing loss, there is therefore direct evidence for inflammation-associated vasospasm causing sensorineural hearing loss.

#### 3.1.6. Presbyacusis

Age-related inflammatory diseases like type II diabetes and cardiovascular disease have been associated with SNHL [31, 32]. In 2012, the first investigation into a correlation of systemic inflammation with SNHL was undertaken in 611 people between 63 and 74 years of age and total white blood cell count, neutrophil count, and C-reactive protein (CRP) levels were found to be associated with SNHL independently of smoking, alcohol, and previous noise exposure. The authors did not forward any explanation for this finding [33]. The results of this study were confirmed in the “Epidemiology of Hearing Loss Study,” which found an association of elevated CRP levels at baseline and future development of SNHL [34].

The theoretic possibility of vasospasm related to arteriosclerotic inflammation is raised by the transient audiovestibular dysfunction with transient hearing loss found as prodromal symptom of stroke affecting the supply area of the anterior inferior cerebellar artery in vertebrobasilar ischemic stroke affecting 8 to 31% of prospectively observed patients including patients with normal diffusion-weighted MRI after the attack [35]. Brown et al. [36, 37] found that cochlear vascular reactivity to topical application of nitroprusside, a vasodilating agent, was less in old mice than in young mice.

Overall, the findings in presbyacusis justify the conjecture of an increased risk of inflammation-associated vasospasm-induced sensorineural hearing loss as a contributory underlying mechanism.

#### 3.1.7. Migraine

The pain of migraine-associated headaches has been linked to the activation of nociceptors by the vasoconstriction-inducing cytokines TNF and IL-1 [38, 39]. The involvement of these two cytokines if they are involved in cytokine-induced and “vasospasm-related” SNHL would therefore make one expect that migraine is associated with “vasospasm-related” SNHL. Migraine has been associated with sudden and transient SNHL where there was cerebral infarction and children were affected [40–42] [43]. The findings are therefore overall showing features consistent with inflammation-associated vasospasm-induced sensorineural hearing loss.

### 3.2. Mechanisms Linking Cytokines to Vasoconstriction

Inflammatory mediator action could explain the role of vasospasm in the pathogenesis of SNHL in all the aforementioned conditions. For many years, the cochlea was considered an “immune-privileged” organ because of the presence of a tight junction-based blood-labyrinth barrier. A number of more recent studies, however, showed that resident macrophages are always present in the cochlear lateral wall as well as in the spiral limbus and the scala tympani side of the basilar membrane, and they are activated by various types of insults, including noise exposure, ischemia, mitochondrial damage, and surgical stress leading to cytokine release including tumour necrosis factor [44].

#### 3.2.1. Tumor Necrosis Factor-Induced Vasospasm and Hearing Loss

TNF-alpha was shown to be effective in decreasing cochlear blood flow at a dose of 5.0 ng/mL. Lower concentrations or placebo treatment did not lead to significant changes. After pretreatment with etanercept, a recombinant human TNF receptor p75 Fc fusion protein, which blocks TNF action, TNF-alpha at a dose of 5.0 ng/mL, no longer led to a change in cochlear blood flow [45].

In a prospective clinical trial in Mongolian gerbils, tumor necrosis factor-alpha administered via continuous 8-day intrathecal flow induced SNHL across all frequencies compared to phosphate-buffered saline delivered intrathecally in controls and tumor necrosis factor-alpha antibody injected intraperitoneally reduced SNHL in the pneumococcal meningitis model [46].

In a prospective observational study, tumor necrosis factor-alpha levels in peripheral venous blood were compared between patients with autoimmune inner ear disease and sudden SNHL compared to controls and steroid responders compared with steroid nonresponders. TNF levels were significantly higher in patients with sudden SNHL, and a high baseline plasma TNF of greater than 18.8 pg/ml had a positive predictive value higher than 97% for a sudden change in hearing threshold [47] and was associated with a lack of response to steroids.

The mechanism by which tumor necrosis factor causes a reduction in cochlear blood flow has been identified and involved contraction of cochlear pericytes which has in the guinea pig model been found to cause a reduction of capillary diameter by 3.6 ± 4.3%, which was reversible by anti-TNF therapy with ethanercept [48].

The effect of TNF on vasoconstriction may be mediated by sphingosine-1-phosphate (S1P) [49].

S1P has been demonstrated to lead to vasoconstriction in cerebral and basilar arteries in vivo in animal models. This effect was shown to be mediated by elevation of intracellular calcium levels by mobilisation from intracellular stores in smooth muscle cells. Ca^2+^ sensitization of the contractile filaments, which frequently involves a rho-kinase, is also an effect of S1P and has been observed in human coronary smooth muscle cells. Inhibition of the rho A/rho-kinase pathway has hereby been shown to inhibit vasoconstriction [50]. It has also been established that the tone of the labyrinthine artery is regulated by S1P [51]. It has thus been shown that S1P could be involved in the pathogenesis of inflammation-induced hearing loss. TNF is also able to impair the stability of endothelial NO synthase mRNA, thus potentially undermining the bioavailability of the vasodilator NO [52].

The question is whether the chronic inflammation leading up to presbyacusis leads to SNHL via cytokine-induced apoptosis of hair cells of the inner ear or recurrent vasospasm in the labyrinthine artery. In addition to causing vasospasm, TNF could trigger programmed cell death (apoptosis) in hair cells of the inner ear and thus cause SNHL. The extrinsic pathway to apoptosis includes TNF alpha, which via TNF receptor-associated death domain activates cysteine-aspartic proteases (caspases)-8 and -10. Caspase-8 then can activate tBid to recruit the help of the intrinsic pathway and can activate caspases-3, -6, and -7. These caspases lead to the execution phase of cell death induction through their protein-cutting activity. Caspase-3 can hereby cause DNA and chromatin damage, rearrange the cytoskeleton, and disrupt intracellular transport [53]. Apoptosis-related hearing loss secondary to long-term exposure to circulating mediators of chronic inflammation including TNF should lead to bilateral SNHL. Studies on the effect of the anti-inflammatory drug dexamethasone (DXM) in vitro using 3-day-old rat organ of Corti explants challenged with tumor necrosis factor-alpha (TNFa) demonstrated the otoprotective capability of DXM against the ototoxic effects of this inflammatory cytokine on auditory hair cells. Cytokine-exposed explants treated with DXM prevented TNFa-induced loss of auditory hair cells and resulted in the downregulation of Bax gene expression with upregulation of both Bcl-2 and Bcl-xl gene expression compared with baseline levels of gene expression in control explants and with the expression levels of the housekeeping gene, *β*-actin. Biopolymer- (i.e., poly(styrene-b-isobutylene-b-styrene); SIBS) eluted DXMb retains its ability to prevent the death of explant auditory hair cells that were exposed to TNFa and to initiate the same patterns of upregulation and downregulation of antiapoptosis- and proapoptosis-related genes (i.e., Bcl-2, Bcl-xl, and Bax) that were observed in TNFa-challenged and TNFa-challenged, DXM-treated organ of Corti explants [54].

#### 3.2.2. IL-1 and Vasospasm-Associated SNHL

An investigation in patients with SAH showed that levels of IL-1 beta but not TNF-alpha were increased and correlated with the later development of vasospasm. IL-1 acts through G-protein-coupled receptors in three ways which cause contraction of vascular smooth muscle [1]:

1. Activation of phospholipase C-generated phosphatidylinositol trisphosphate causes calcium release which leads to myosin light chain kinase activation leading to myosin light chain activation and calcium-dependent vasospasm. Phosphorylation of myosin light chain (MLC) by MLC kinase is one of the most important steps for vascular smooth muscle contraction.
2. Activation of protein kinase C (PKC) through phospholipase C-generated diacylglycerol (from phosphatidylinositol (4,5) bisphosphate) can act through phosphorylation, hence activating the myosin light chain kinase. Prolonged contraction in cerebral vasospasm for up to two weeks can be mediated by this mechanism.
3. PKC activation regulates MLC phosphorylation through activation of rho-kinase and the myosin-binding subunit (MBS) of MLC phosphatase (MLCPh).

A secondary pathway for vascular smooth muscle contraction that is not directly dependent on Ca^2+^ concentration, but rather mediating Ca^2+^ sensitization, is the rho A/rho-kinase pathway. In cultured human coronary vascular smooth muscle cells, inflammatory stimuli, such as IL-1 beta, increase rho-kinase expression (at both mRNA and protein levels) and function [55].

**Figure 2:**
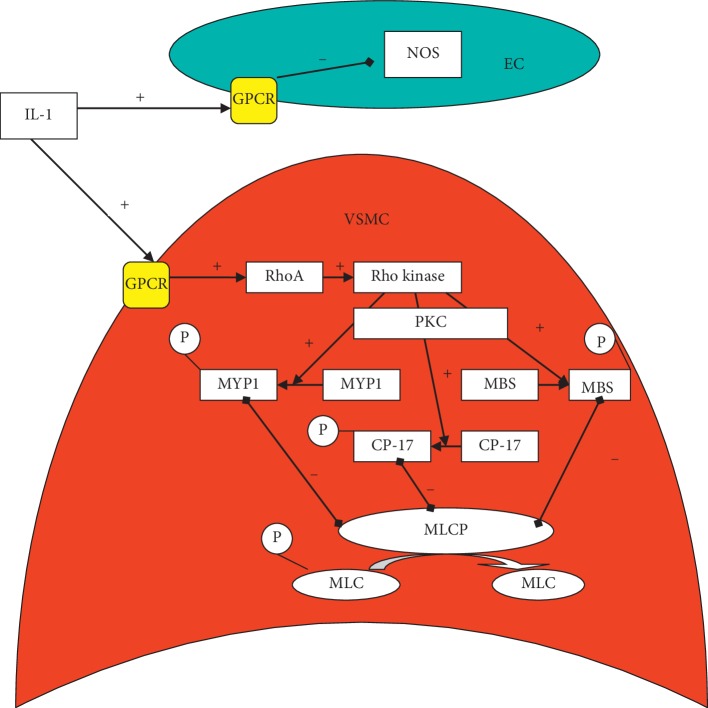
Mechanism of action of interleukin-1 (IL-1) via G-protein coupled receptors (GPCR) on reduction of nitric oxide (NO) production by reducing nitric oxide synthase (NOS) activity in the vascular endothelial cell (EC) and by activation of the enzyme Rho kinase via RhoA in the vascular smooth muscle cell to activate by phosphorylation through protein kinase C (PKC) myosine phosphatase targeting subunit-1 (MYPT1), protein kinase C-potentiated phosphatase inhibitor-17 (CPI-17 and myosine binding subunit (MBS)), which all three inhibit the myosine light chain phosphatase (MLCP) which dephosphorylates the myosine light chain, a process, which is necessary for smooth muscle relaxation. This process thus causes vasoconstriction.

Both endothelial NO production and NO-mediated signaling in vascular smooth muscle cells (VSMC) are targets and effectors of the rho A/rho-kinase pathway. In endothelial cells (ECs), the rho A/rho-kinase pathway negatively regulates NO production [56]. In addition, in response to contractile stimuli, the small GTPase rho A activates its downstream effector rho-kinase which, in turn, promotes contraction via myosin light chain phosphatase (MLCP) inhibition. Rho-kinase activity is enhanced by binding to the active GTP-bound rho A. Experiments were performed to examine whether rho-kinase is upregulated at the spastic site and how it induces VSMC hypercontraction if it is upregulated. Reverse transcriptase polymerase chain reaction analysis demonstrated that the expression of rho-kinase mRNA and, to a lesser extent, that of *RhoA* mRNA were upregulated in the spastic site than the control coronary site. Rho A/rho-kinase-mediated MLCP inhibition occurs mainly by phosphorylation and inhibition of MYPT-1, the regulatory subunit of MLCP, or by CPI-17-mediated inhibition of the catalytic subunit of MLCP. Rho-kinase hereby phosphorylates MBS, which results in the inhibition of MLCPh [57]. These results indicate that rho-kinase is upregulated at the spastic site and plays a key role in inducing VSMC hypercontraction by inhibiting MLCP through MYPT-1 phosphorylation. The reduced MLCPh activity leads to increased phosphorylation and hence contractility of the MLC resulting in calcium-independent vasospasm (See Figure 2). Investigations in a porcine model revealed that hydroxyfasudil, a specific rho-kinase inhibitor, exerted an inhibitory effect on vasospasm both in vitro and in vivo. Western blot analysis showed that, during serotonin-induced contractions, the extent of phosphorylation of the MBS was significantly greater in the spastic than in the control segment. There was a highly significant correlation between the extent of MBS phosphorylation and contractions. The potential of fasudil in prevention and treatment of vasospasm-associated SNHL was demonstrated in an experiment with the small modiolar artery isolated from temporal bone of gerbils in which it prevented endothelin-1-induced contraction and Ca^2+^ sensitization [58].

Endothelin-1 is one way of action of both TNF and IL-1 on vasospasm-induced hearing loss. Only ET-1 is produced in cerebral endothelium and mediates cerebral vasoconstriction via endothelin-A (ETA) receptors. ETA is localized in vascular smooth muscle cells, and stimulation leads to increase in intracellular calcium concentrations leading to vasoconstriction. ET-1 expression is enhanced by IL-1 and TNF and can be inhibited by NO, nitric oxide donor drugs, and dilator prostanoids via an increase in cellular cGMP. NO can produce relaxation of some blood vessels from some species via activation of potassium channels [1].

#### 3.2.3. Cytokine Gene Polymorphisms and SNHL

Should cytokines have a role in SNHL one would expect that cytokine gene polymorphisms which are associated with differences in cytokine production differ between patients prone to hearing loss and those who are not. This is in fact the case.

A significant difference in carriage of both the IL-1*β* − 511 T allele and the IL-1*β* + 3953 T allele was observed between sudden SNHL and controls. This suggests that the IL-1*β* − 511 and +3953 loci may play an important role in the etiopathogenesis of SSNHL [59].

Genetic linkage studies have investigated the association of polymorphisms of the TNF-alpha and TNF-beta genes with sudden SNHL comparing 97 SSNHL patients and 587 controls and found that the TNF-beta +252 A− ⟶ G polymorphism which is associated with increase in production of this cytokine was significantly increased in patients with SSNHL [60], a locus, which had previously been linked to cerebral infarction [61].

### 3.3. Treatment and Prevention of Vasospasm-Induced SNHL

#### 3.3.1. Calcium Antagonists

*(1) Calcium-Channel Blockers*. As illustrated in anti-phospholipid antibody syndrome, an autoimmune inflammatory syndrome, which is associated with cerebral vasoconstriction syndrome, centrally acting calcium antagonists like nimedipine, can reduce symptoms of cerebral vasoconstriction, which include hearing loss [62].

Blockade of L-type voltage-gated Ca^2+^ channels protected against NIHL in mice and in guinea pigs, and blockade of T-type voltage-gated Ca^2+^ channels was effective in mice. Also consistent with a contribution of calcium-mediated events in hair cell damage, application of the calcineurin inhibitor FK506 attenuated NIHL in guinea pigs [63–66].

*(2) Magnesium*. An agent that can lead to vasodilatation by virtue of antagonism of calcium-dependent vascular smooth muscle contraction is magnesium. It seems to act through a variety of pathways. Experiments in laboratory animals demonstrated that as magnesium decreases, there is a greater secretion of catecholamines and prostaglandins, subsequently leading to a reduction of the blood flow because of vasoconstriction in the inner ear and with it, a higher risk of energetic depletion in the hair cells. Increased magnesium intake can thereby improve inner ear microcirculation. The muscle tone of the vessels diminishes with higher concentrations of intracellular free magnesium, and their reactivity to vasoactive substances decreases [67–69].

In addition to the well-characterized effects on vasodilation, biochemical effects of magnesium include modulation of calcium channel permeability, influx of calcium into cochlear hair cells, and glutamate release [70, 71].

The first evidence for the importance of magnesium in prevention of SNHL was gained in observational studies of magnesium deficiency in rats and guinea pigs exposed to noise. Magnesium deficiency increased susceptibility to NIHL in rats [72] and guinea pigs [73]. Subsequent experiments then confirmed the protective effect of magnesium in prevention of SNHL in the guinea pig model [74–76].

The results of magnesium in animal experiments have have been confirmed in 3 randomised controlled trials [77–79] summarized in Table 2 which confirmed their applicability in humans and that magnesium has the potential to prevent noise-induced SNHL in humans. When measured it was not serum magnesium levels, which were different between groups, but the magnesium levels in red blood cells and monocytes or lymphocytes [78, 79].

#### 3.3.2. Anti-Inflammatory Therapies

*(1) Etanercept*. Etanercept is a widely used biological agent in the treatment of autoimmune diseases such as rheumatoid arthritis, psoriasis, and others by targeting TNF-*α*. This and other anticytokine therapies, which show promise in prevention or treatment of inflammation-induced SNHL, have been reviewed previously [80], and the results of this review were that etanercept can mitigate the inflammation and hearing loss (HL) in labyrinthitis induced by keyhole limpet hemocyanin (KLH) in mice. In a guinea pig model of immune-mediated HL, within 3–5 days of KLH insult, the labyrinth developed features of inflammation associated with HL. Both the systemic treatment with etanercept and long-term infusion of etanercept (for 7 days) into the scala tympani significantly protected the labyrinth against KLH-induced inflammation and the development of HL. Systemic administration of etanercept after loud noise exposure (106 dB SPL, 30 min) significantly attenuated NIHL. The microcirculatory analysis of cochlea showed significant increase in cochlear blood flow in strial capillary segments. Moreover, significant hearing improvement was observed in etanercept-treated groups versus controls. The TNF-*α* inhibitor etanercept has hereby been shown to reduce noise-induced threshold shifts [81]. A very small randomised controlled trial in autoimmune SNHL in humans did not show any benefit, but the small sample size (*n* = 17 in total) means that a clinically significant difference may have been missed [82].

*(2) Steroids*. The anti-inflammatory and proresolution glucocorticoid dexamethasone (DEXA), which is able to reduce TNF production, has been investigated after systemic, intratympanic, and combined delivery in sudden and noise-induced SNHL but with mixed results as shown in the following four meta analyses [83–86] (Table 3).

*(3) Statins*. Statins are inhibitors of the 3-hydroxy-3-methyl-glutaryl-coenzyme A reductase, which is a key enzyme of the mevalonate pathway, the metabolic pathway that produces cholesterol and other isoprenoids. It also has a direct effect on rho-kinase activity and hence the predisposition to vasospasm. This effect is mediated through several different mechanisms: because statins inhibit an early step in the cholesterol biosynthetic pathway, they also inhibit the synthesis of isoprenoids such as farnesylpyrophosphate (FPP) and geranylgeranylpyrophosphate (GGPP), which are important posttranslational lipid attachments for intracellular signaling molecules such as the rho GTPases. Treatment with statins prevents endothelial nitric oxide synthase (eNOS) downregulation by prolonging the half-life of eNOS mRNA. The prolongation of half-life of eNOS mRNA by statins is reversed by GGPP, but not FPP, suggesting the involvement of small GTPases such as rho GTPase in this process. Indeed, decrease in rho GTPase responses as a consequence of statin treatment increases the production and bioavailability of endothelium-derived NO. The mechanism involves, in part, rho/rho-kinase- (ROCK-) mediated changes in the actin cytoskeleton, which leads to decreases in eNOS mRNA stability. In addition to increase in eNOS expression by statins, statins can also rapidly induce the phosphorylation and activation of eNOS via the phosphatidylinositol-3 kinase (PI3K)/protein kinase Akt pathway [87]. Inhibition of mevalonate synthesis by statins decreases geranylgeranyl pyrophosphate and prevents the geranylgeranylation of rho and therefore its activation of rho-kinase (ROCK) [88]. It has been reported that atorvastatin reduces inducible nitric oxide synthase expression in native endothelial cells in situ and the underlying mechanism is associated with inhibition of tumor necrosis factor-*α* and interferon-*γ* expression [89]. In rats, atorvastatin was able to prevent noise-induced SNHL [90]. The same was found in mice [91] and more recently in guinea pigs [92]. In a prospective study of 84 hyperlipidemic patients and audiological assessment before and 6 months after commencement of a statin, a significant reduction of the hearing threshold at 6000 Hz was found and a significantly improved speech discrimination as well as a significant reduction in tinnitus frequency, duration, and severity and degree of annoyance [93].

This result confirmed a previous study: The Blue Mountains Hearing Study is a population-based survey of age-related hearing loss which aimed to assess associations between dietary intake of fats (saturated and monounsaturated fats and cholesterol) and certain food groups (butter, margarine, and nuts) with the prevalence, incidence, and progression of age-related hearing loss. It also investigated the link between serum lipids and cholesterol-lowering medication (statins) and hearing loss. Among persons self-reporting statin use (*n* = 274), a 48% reduced odds of prevalent hearing loss was observed after multivariable adjustment (OR = 0.52 (95% CI = 0.29–0.93)) [94].

On the other hand, a population-based study in Taiwan showed a slight but significant association of previous statin use and SNHL (27% with versus 21% without previous statin use, OR 1.36 (95% CI: 1.18 to 1.57)) [95].

The only randomised double-blind clinical trial in this area was geared to investigate the impact of statins on presbyacusis. It was very small with a total of 50 patients. Pure-tone audiometry and tinnitus evaluation at enrolment and after 7 and 13 months of atorvastatin did not show any impact on hearing thresholds or tinnitus [96].

*(4) Oral Curcumin*. Curcumin, a compound isolated from the plant *Curcuma longa*, is the principal curcuminoid of turmeric, a member of the ginger family. It has been shown to reduce vascular inflammation and the related vasospasm in cerebral blood vessels in animal models of subarachnoid haemorrhage [97, 98].

It is therefore not surprising that oral curcumin in a highly bioavailable preparation has been shown to reduce noise-induced SNHL in a murine model in a dose-dependent fashion [99].

One mechanism may be its capacity for reduction of TNF expression [100] which is probably mediated by inhibition of epigenetic activation of cytokine expression through inhibition of histone deacetylase expression [101].

*(5) Phosphodiesterase-5 Inhibitors*. Phosphodiesterase-5 inhibitors are potent vasodilators and can relieve vasospasm. It is therefore conceivable that they could reduce or prevent inflammatory vasospasm-related hearing loss. The mechanism of action involves causing increased bioavailability of cGMP, a potent endogenous vasodilator: When nitric oxide (NO) forms in the body, it avidly binds to the heme moiety of the enzyme guanylyl cyclase, which is found in vascular smooth muscle cells. NO is formed by vascular endothelium and diffuses into the vascular smooth muscle cells adjacent to the endothelium where it binds to and activates guanylyl cyclase. This enzyme catalyzes the dephosphorylation of GTP to cGMP, which serves as a second messenger particularly for signaling smooth muscle relaxation. Cyclic GMP induces smooth muscle relaxation by multiple mechanisms including

1. Increasing intracellular cGMP, which inhibits calcium entry into the cell, and decreasing intracellular calcium concentrations
2. Activating K^+^ channels, which leads to hyperpolarization and relaxation
3. Stimulating a cGMP-dependent protein kinase that activates myosin light chain phosphatase, the enzyme that dephosphorylates myosin light chains, which leads to smooth muscle relaxation

Because of the central role of cGMP in NO-mediated vasodilation, drugs that inhibit the breakdown of cGMP (cGMP-dependent phosphodiesterase inhibitors) are used to enhance NO-mediated vasodilation.

Experiments in the mouse model of noise-induced SNHL demonstrated that Pde5 inhibition protected against NIHL [102]. Pde5 inhibition has also been clinically beneficial in reversing deficits in stroke patients [103].

There have been concerns about an association of phosphodiesterase-5 inhibitor use and SNHL but this can be attributed to a confounding association of erectile dysfunction and hearing loss. Erectile dysfunction is the most common indication for phosphodiesterase-5 inhibition. Erectile dysfunction is liked to SNHL because both can be due to vascular dysfunction or vasospasm in the supplying artery in arteriosclerosis [104–107].

## 4. Discussion

Inflammation is a normal adaptive response aimed at restoring tissue functionality and homeostasis after infection and tissue injury, and suppressing it could have unintended negative consequences. Previous authors recognized the potentially important role of inflammation in causing age, noise, and drug-induced acquired SNHL but merely proposed to reduce the inflammatory response as a strategy to reduce SNHL [44]. This approach may not be rapidly and comprehensively effective if only directed at cochlear inflammation and not taking into account the importance of rapidly reversing vasospasm in order to reduce ischemic damage to the hair cells and other vital structures of the cochlea including targeting the specific mediators involved in vasospasm.

The evidence for a link between inflammation-induced vasospasm and acquired SNHL revealed in this review can be categorized as indicating a risk, as showing features or directness for the conditions identified above (see Table 4):

In conditions like bacterial meningitis, there is some hearing loss and there is some indirect evidence that there is vasospasm in bacterial meningitis. Further research needs to link vasospasm with hearing loss in prospective observational studies focussing on cochlear blood supply.

Similarly in traumatic brain injury, there is hearing loss in brain injury and there is vasospasm in brain injury, but the association between brain injury and vasospasm-mediated SNHL is only inferred from these two separate sets of findings, without being actually proven in any study.

Inhibition of rho-associated kinases by agents like fasudil needs to be explored in all patients at risk of vasospasm associated hearing loss or with established acquired SNHL [108, 109].

Future studies need to explore substances known to increase cochlear blood flow in ex vivo experiments, and these include herbal drugs used by traditional herbal medicine to treat hearing loss like *Ginkgo biloba*-derived quercetin, rosmarinic acid, and curcumin, the application of which has been shown to dilate cochlear vasculature and improve cochlear blood flow as measured by Doppler flowmetry in the guinea pig ex vivo model [110, 111].

Tinnitus has been associated with vasospasm in subarachnoid haemorrhage [112] and migraine has been associated with both vasospasm and tinnitus [41, 42], and vertigo is associated with SNHL in vascular compromise [113].

Vascular inflammation may not just be reduced in the labyrinthine artery by the modes of treatment explored in this review but other vascular beds as well, and future randomised controlled trials should incorporate other vestibular disorders including vertigo and tinnitus and vascular headaches as secondary outcomes as well as heart disease, stroke, vascular dementia, peripheral vascular disease, and their incidence could be monitored during long term follow-up of patients enrolled.

## 5. Conclusions

There is mainly indirect evidence for vasospasm-associated SNHL in most forms of systemic or injury- or infection-induced local vascular inflammation. This opens up avenues in prevention and treatment of vascular and systemic inflammation as well as vasospasm itself as a way to prevent and treat most forms of acquired SNHL. Future research needs to investigate interventions antagonising vasospasm and vasospasm-inducing proinflammatory cytokines and their production in randomised controlled trials of prevention and treatment of acquired SNHL. Prime candidates for interventions are hereby inflammasome inhibitors and vasospasm-reducing drugs like nitric oxide donors, rho-kinase inhibitors and magnesium which have the potential to reduce SNHL in meningitis, exposure to noise, brain injury, arteriosclerosis, and advanced age-related and autoimmune disease-related inflammation.

## Figures and Tables

**Table 1:** Clinical manifestations, indicators of disease severity, and SNHL in bacterial meningitis (data from [10]).

	Cases with SNHL after bacterial meningitis (*n* = 41)	Controls without SNHL after bacterial meningitis (*n* = 129)	*P* value	Odds ratio (95% CI)
Signs of shock on admission	13	48	0.65	0.78 (0.35–1.76)
Mechanical ventilation	2	16	0.24	0.36 (0.05–1.76)
Seizures	10	29	0.96	1.11 (0.45–2.71)
Focal neurological signs	16	9	<0.001	9.0(3.11–27.7)

**Table 2:** Evidence for effectiveness of magnesium in prevention of noise-induced SNHL from randomised controlled trials in humans.

Design	Number of participants	Age of participants	Intervention	Outcome	Critical appraisal	Reference
Placebo-controlled double blind	28 participants in total, number per group unknown	22 to 75 years of age, mean 53 years	6.7 mmol of magnesium aspartate orally once a day	Improvement of sudden SNHL excluding noise, mumps, Meniere's disease, and traumatic SNHL: more patients with improved hearing and a greater improvement in those with SSNHL were noted in the magnesium treated group across all frequencies assessed	The investigation did not report method of randomisation, number randomised into each group, and number of patients with improved hearing for each frequency	[77]

Placebo-controlled double blind	150 participants in each group	17.7 to 18.5 years old	6.7 mmol of magnesium aspartate orally once a day	Noise-induced permanent hearing threshold shifts in 11.2% in the magnesium group versus 21.5% in the placebo group (*p*=0.03)	The method of randomisation was unclear, and the duration of prophylactic treatment and timing of measurement were not specified	[78]

Placebo-controlled double blind	215 participants, 105 in the magnesium group, and 110 in the placebo group	18-year-old men	6.7 mmol of magnesium aspartate orally once a day	There was a noise-induced pure tone audiometry threshold shift (>25 dB at 3 to 8 kHz) in 11.2% (both ears) of the magnesium group and 21.5% in the left ear and 28.5% in the right ear in the placebo group	The method of randomisation was unclear, and the duration of prophylactic treatment and timing of sampling were not specified	[79]

**Table 3:** Meta-analyses of randomised controlled trials in humans to treat SNHL with steroids.

Design	Number of participants/number of randomised controlled trials	Age of participants	Intervention	Outcome	Critical appraisal	Reference
Systematic review with meta-analysis of randomised controlled trials	1394/14	Unknown	Combined intratympanic and systemic use of steroids as a first-line treatment for sudden SNHL	The proportion of patients with hearing improvement as the outcome measure was observed in 13 studies, which resulted in an odds ratio (OR) of 2.50 (95% confidence interval (CI): 1.95–2.1)	The differences in treatment regimes or unclear treatment invalidate the pooling of results	[83]

Systematic review and meta-analysis of randomised controlled trials	416/8	Mean age 47 to 60 years	Isolated intratympanic dexamethasone for sudden SNHL	Pure-tone audiogram improvement criterion did not reach statistical significance (OR 5 0.39, CrI 5 0.11–1.27)	Large heterogeneity was noted among these studies	[84]

Systematic review and meta-analysis of randomised, controlled trials	203 /5	Unknown overall age distribution	Intratympanic steroid therapy as a salvage treatment for sudden SNHL after failure of conventional therapy	The meta-analysis data were derived from 5RCTs of 102 patients in the ITS group and 101 control subjects; the mean difference and 95% CI of the PTA improvement (indB) were 7.43 and 4.25–10.60, respectively	The authors suspected that the small number of trials (5) available for their meta-analysis was due to publication bias with studies reporting nonsignificant results under represented	[85]

Systematic review and meta-analysis of randomised, controlled trials	1166/15	Unknown	Treatment of sudden SNHL	Three articles (181 subjects), steroid versus placebo analysis: OR = 1.52 (95% confidence interval (CI): 0.83–2.77); six articles (702 subjects) in systemic versus intratympanic steroids analysis (OR 1.14 (95% CI: 0.82–1.59)); six articles (283 subjects): salvage treatment analysis (OR: 6.04 (95% CI: 3.26–11.2))	Numbers are small in the subgroup analyses shown; a clinically significant effect may have been missed	[86]

**Table 4:** Directness of evidence for a role of inflammation-induced vasospasm in conditions associated with acquired SNHL.

Condition associated with SNHL	Evidence for vasospasm	Evidence of link of SNHL to vasoconstrictors	Evidence for response to anti-inflammatory treatment	Evidence for treatment response to vasodilators
Autoimmune	Features	Features	Features	Not investigated
Bacterial meningitis	Features	Features	Features	Features
Phonic injury	Direct	Direct	Direct	Not investigated
Subarachnoid haemorrhage	Features	Features	Features	Not investigated
Brain trauma	Increased risk	No evidence	No evidence	Not investigated
Presbyacusis	No evidence	No evidence	No evidence	Not investigated
Migraine	Features	Not investigated	Not investigated	Not investigated
Sudden SNHL	Direct	Not investigated	Direct	Direct

## Abbreviations

AICA: Anterior inferior cerebellar artery
GPCR: G-protein coupled receptors
CD: Capillary density
CPI-17: Protein kinase C-potentiated phosphatase inhibitor-17
FPP: Farnesylpyrophosphate
GGPP: Geranylgeranylpyrophosphate
HL: Hearing loss
IL-1: Interleukin-1
MBS: Myosine-binding subunit
MLC: Myosine light chain
MLCPh: Myosine light chain phosphatase
MYPT1: Myosine phosphatase targeting subunit-1
NIHL: Noise-induced hearing loss
NO: Nitric oxide
NOS: Nitric oxide synthase
PKC: Protein kinase C
PORH: Postocclusive reactive hyperaemia
S1P: Sphingosine-1-phosphate
SNHL: Sensorineural hearing loss
TNF: Tumor necrosis factor.
